# Microwave-assisted direct synthesis of butene from high-selectivity methane

**DOI:** 10.1098/rsos.171367

**Published:** 2017-12-20

**Authors:** Yi-heng Lu, Kang Li, Yu-wei Lu

**Affiliations:** 1 School of Chemical Engineering, Anhui University of Science and Technology, 232001 Huainai, People's Republic of China; 2 Laboratoire de Chimie Physique, Université de Paris Sud, 91405, Orsay Cedex, France

**Keywords:** methane, microwave heating, high selectivity, butane

## Abstract

Methane was directly converted to butene liquid fuel by microwave-induced non-oxidative catalytic dehydrogenation under 0.1–0.2 MPa. The results show that, under microwave heating in a two-stage fixed-bed reactor, in which nickel powder and NiO*_x_*–MoO_y_/SiO_2_ are used as the catalyst, the methane–hydrogen mixture is used as the raw material, with no acetylene detected. The methane conversion is more than 73.2%, and the selectivity of methane to butene is 99.0%. Increasing the hydrogen/methane feed volume ratio increases methane conversion and selectivity. Gas chromatography/electron impact ionization/mass spectrometry chromatographic analysis showed that the liquid fuel produced by methane dehydrogenation oligomerization contained 89.44% of butene, and the rest was acetic acid, ethanol, butenol and butyric acid, and the content was 1.0–3.0 wt%.

## 1. Introduction

After ethylene and propylene, butene is an important intermediate in chemical production; butene is the raw material of butadiene, and butadiene is the main raw material for the production of synthetic rubber, such as styrene butadiene rubber, butadiene rubber, nitrile rubber and chloroprene rubber. With the development of styrene plastics, the use of styrene and butadiene co-polymer can produce widely used resins such as acrylonitrile-butadiene-styrene copolymer, styrene-butadiene-styrene block copolymer, butadiene-styrene copolymer, and methyl methacrylate-butadiene-styrene terpolymer. At present, butene is generally obtained by petroleum cracking; however, with ever-decreasing and depleted oil reserves, potential alternatives will come from the dehydrogenation coupling of methane from natural gas or biogas. Because the main component of natural gas is methane, it is very stable, and the formation of ethylene, acetylene and high-hydrocarbon aromatics requires a reaction temperature of 1273 K or higher [1–4]. Although the high reaction temperature is favourable for high conversion rates, e.g. methane conversion of up to 50%, higher temperatures will cause the product to decompose into carbon, with high energy consumption and poor environmental outcomes. Over the past few decades, environmentally friendly methane dehydrogenation studies have attracted widespread attention, such as the study of the use of microwave and plasma discharge technology, with the former becoming gradually popular in industrial heating.

The popular research topics mainly use microwave plasma technology to convert methane directly into higher hydrocarbons to improve the selectivity and yield of the desired products. At room temperature and atmospheric pressure, acetylene was synthesized from methane via DC pulsed discharge, with a selectivity of 95%, a conversion rate of 16–52% and the use of coexisting oxygen to remove carbon deposition and stable discharge state [5]. Under the action of Fe, Ni, Co and Cu, for the microwave oxidative coupling of methane, the selectivity to ethylene is 29.8%, the ethane is 10%, the acetylene is 49.9%, the methane conversion is 54.9%, the total flow is 125 ml min^−1^, and Co/ZSM-5 catalyst has a methane/oxygen ratio of 4 : 1 v/v [6]. Under the corona discharge, methane decomposition was found to produce no CO*x* hydrogen, the selectivity of hydrogen was 56%, and the conversion of methane was 31% [7]. In a study of Ni-based bimetallic heterogeneous catalyst for energy and the environment, such as environmental remediation, catalysis occurs because of its adjustable chemical/physical properties via the composition of the bimetallic system, the preparation method and their morphological structure.

From the literature, the basic understanding of bimetallic systems and their catalytic behaviour, including nickel-based bimetallic catalyst, is mainly for the chemical and electrochemical processes in catalytic reforming, dehydrogenation, hydrogenation and electro-catalytic reactions [8]. A study on the interaction of microwave catalysts in hydrocarbon reforming [9] and the role of microwave in heterogeneous catalytic system [10] were cited. The H-ZSM-5 catalyst has a high activity for the direct conversion of ethylene to propylene at 723 K; the conversion of ethylene is 58%, and the selectivities of propylene and butene are 42% and 21%, respectively [11]. The preparation of acetylene via methane microwave plasma coupling [12,13] was cited, in which process methane might by activated by microwave plasma [14] or dielectric barrier discharge plasma [15]. The effect of frequency on the catalytic oligomerization of methane by microwave heating [16] and methane through the microwave plasma dimer were studied. Ni-AlSBA-15 mesoporous catalysts enable the highly active and stable homogeneous oligomerization of ethylene [17]. Ni-containing zeolite has a property of low polymerization of ethylene, as demonstrated by studies in chemistry and infrared spectroscopy [18]. Catalytic ethylene dimerization and oligomerization with nickel complexes containing P, N-chelating ligands [19] and the oligomerization of methane by microwave heating [20] were demonstrated.

Regarding the dehydrogenation of methane to produce high-carbon hydrocarbons, many reports in the literature involve the use of microwave or plasma discharge technology; by contrast, the use of inexpensive raw materials of methane of high selectivity and direct preparation of butane has never been reported. We report here the technology using methane/hydrogen (1 : 5 v/v) mixed gas, nickel powder and NiO*_x_*–MoO*_y_*/SiO_2_ as the catalyst and microwave excitation for direct liquid fuel synthesis. The methane conversion in the ‘two-stage fixed bed’ is greater than 73.2%, and the butene selectivity is more than 99%. Direct synthesis of liquid products through gas chromatography/electron impact ionization/mass spectrometry (GC/EI/MS) showed that the content of butene was 89.44% and the remaining liquid components were ethanol, acetic acid, butenol and butyric acid of contents of 1.0–3.0 wt%. The high selectivity of methane has significant novelty, and this technology has industrial application prospects.

## 2. Experimental procedure

### 2.1. Materials

Ammonium heptamolybdate tetrahydrate (99.98% pure), nickel (II) nitrate hexahydrate (99.0% pure) and silica (99.0% pure) all are of analytical grade. Nickel powder, 99.999% of purity, grey irregular powder was purchased from the China Metallurgical Research Institute. Methane (purity 99.9%) and hydrogen (purity 99.999%) were purchased from the Nanjing Special Gas Co., Ltd.

### 2.2. Instrumentation

The microwave oven model is G70F20N3P-2s, with power of 700 W, produced by the Guangzhou Galanz Electric Co., Ltd. The methane dehydrogenation polymerization liquid product has the appearance of a colourless oily liquid that is easy to volatilize. X-ray diffractometer (XRD) analysis was performed using a TTR-III type XRD manufactured by the Rigaku Corporation, having a Cu-K*α* source (wavelength *λ* = 1.54178 Å) operated at a voltage of 40 kV and a current of 200 mA; the scanning step is 0.02°, the scanning speed is 8° min^−1^, and the scanning range 2*θ* is 5–80°.

The gas chromatograph model GC-2060 was produced by the Shandong Huifen Co., Ltd.; a six-way valve injection was used, the carrier gas was hydrogen, the reaction gas product was analysed by the thermal conductivity, thermal conductivity detector (TCD), determination, a column for the stainless-steel column was used, the fixed phase was GDX-502, 80–100 mesh, the column outer diameter was 3 m × Ф3 mm (length × inner diameter), the carrier gas I was 0.08 MPa, the carrier gas II was 0.05 MPa, the column temperature was 60°C, the gasification (injection) temperature was 70°C, the thermal conductivity (detector) was 100°C, and the bridge current was 80 mA using the N2000 chromatographic workstation, with the area normalization method used to calculate the component content.

The resulting liquid product was analysed using a Thermo Scientific Company Q Exactive GC gas chromatography time-of-flight mass spectrometer (EI) instrument (Orbitrap). The column was base peak integration 60 m × 0.25 mm, 0.25 µm, the carrier gas was He, the control mode was 1 ml s^−1^, and the splitting ratio was 100 : 1. The furnace temperature was initially at 30°C, increased at a rate of 2°C min^−1^, and then kept at 210°C for 5 min (*m/z*: 30–400). The automatic gain control target was 1 × 10^6^ with resolution of 120 000, the electron bombardment voltage was 70 eV, the sample injection volume was 1 μl, and the ion source temperature is 250°C; in the electro-spray positive ion mode EI^+^, the emission current was 50 mA, and interface temperature was 250°C. Scanning electron microscopy (SEM) was analysed using a Gemini SEM 500 Schottky field emission SEM. The LABRAM-HR800 Raman spectrometer of the French JY company was used to perform Raman spectroscopy using the following parameters: an excitation wavelength of the argon ion laser of 514.5 nm, a spot diameter of approximately 10 μm, a sample laser power of less than 1 mW, and a back-scattering configuration.

### 2.3. Experimental setup

**Figure 1.**
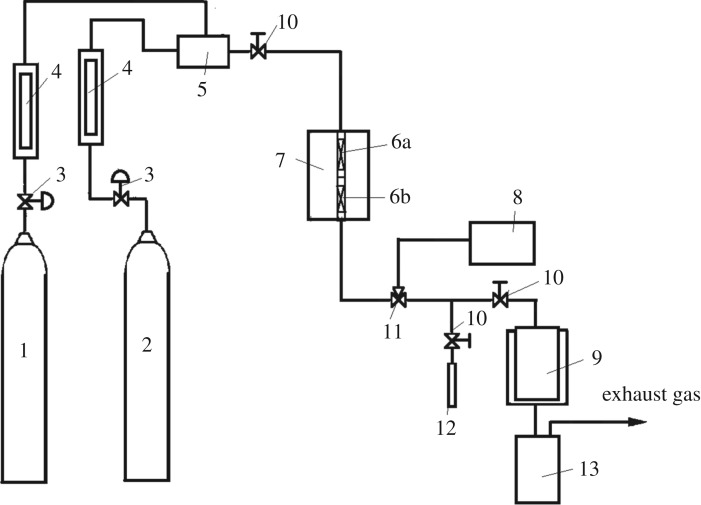
Schematic diagram of the experimental process for the production of butene from methane. (1) methane; (2) hydrogen; (3) pressure reducing valve; (4) flow meter; (5) mixer; (6a) one-stage fixed bed; (6b) two-stage fixed bed; (7) microwave reactor; (8) gas chromatograph; (9) condensing cooler; (10) regulating valve; (11) six-way valve; (12) soapflow meter; (13) liquid product storage tank.

The experimental device diagram of methane synthesis of butene is shown in figure 1. The device is composed of three parts: a feed gas flow control device, a microwave reactor and a reaction product condensed gas–liquid detection system. The microwave frequency is 2.45 GHz, and the power is 700 W. The microwave reactor was composed of a cylindrical quartz glass tube having a length of 200 mm and an inner diameter of 10 mm. The first stage of the reactor was filled with pure nickel powder catalyst and connected to the second stage of the reactor filled with NiO*_x_*–MoO*_y_*/SiO_2_ composite catalyst.

### 2.4. Preparation of the nickel–molybdenum/SiO_2_ catalyst (impregnation method)

First, 4.460 g (0.0153 mol) of nickel nitrate hexahydrate and 1.656 g (Mo: 0.0094 mol) of ammonium heptamolybdatetetrahydrate were dissolved in 8 ml of deionized water to obtain a metal salt aqueous solution and then mixed and stirred for 30 min at room temperature. The measured 8.212 g of silica (SiO_2_) was added to the aqueous solution of metal salt, immersed at 80°C for 16 h, dehydrated at 90°C for 8 h, and then dried at 120°C for 8 h; subsequently, it was calcined at 500°C for 8 h in air atmosphere to remove water, oxygen and nitrogen dioxide. The nickel oxide and molybdenum oxide and its silica carrier catalyst NiO–NiMoO_4_ (0.6277: 1.0 mol)/SiO_2_ were obtained, and then the catalyst was reduced at 700°C in a hydrogen atmosphere for 1 h. With the diameter of the catalyst particles of approximately 200 nm and the hydrogen feed flow rate of 60 ml min^−1^, the NiO*_x_*–MoO*_y_*_(*x*=0–1,*y*=0–3)_/SiO_2_ catalyst was obtained, with the nickel and molybdenum content of 9(wt%) each.

### 2.5. Methane microwave catalytic production of the butene test

In the microwave reactor, the methane hydrogen mixed gas (CH_4_:H_2 _= 1 : 5 v/v) was passed through a Teflon tube. First, the gas was flowed through the first stage of the reactor (quartz tube) filled with 1.000 g of pure Ni powder catalyst; the reaction intermediate product gas continues to flow through the second stage of the reactor (quartz tube), and then 0.500 g of the NiO*_x_*–MoO*_y_*/SiO_2_ composite catalyst powder was added to the tube, followed by microwave heating at 700 W for 30 min, with the methane–hydrogen mixed gas inlet pressure of 0.1–0.2 MPa, the inlet flow rate of methane of 10 ml mi^−1^ and that of hydrogen of 50 ml min^−1^. When the product gas flows through the second stage of the reactor (quartz tube), the reaction gas is collected by a six-way valve and then analysed online by a GC/TCD. Next, the product mixture is passed into a cold trap (−30°C) containing frozen ethanol solution; after cooling, 1.01 g of colourless liquid product was collected and analysed using GC/EI/MS, and then the non-condensed gas was vented.

## 3. Results and discussion

### 3.1. X-ray diffraction determination of the nickel powder

**Figure 2.**
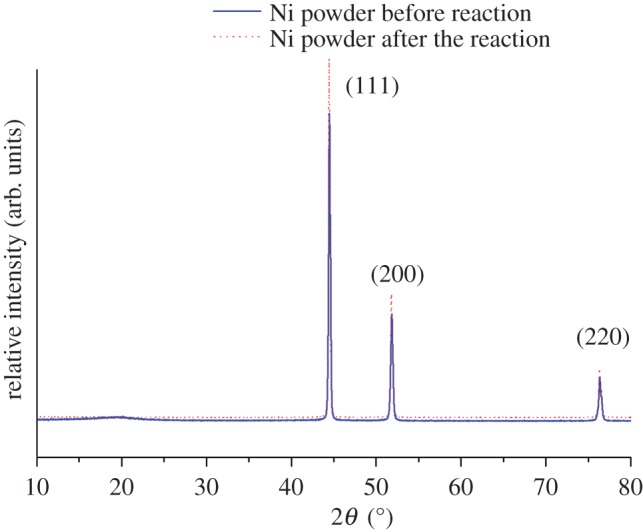
XRD patterns of the Ni powder in the first stage of the reactor.

The crystal structure and phase analysis of the Ni powder in the first stage of the reactor was characterized by powder XRD. The XRD patterns of the Ni powder shown in figure 2 were indexed to monoclinic Ni according to the JCPDS database no. 04-0850 [21]. The average crystallite size of Ni sample was calculated by using the Debye–Scherrer formula given in equation (3.1):

3.1 $$d=\frac{k\lambda }{\beta \;\cos\theta },$$

where *d* is the crystallite size, *k* is 0.89 (CuK), *λ* is the wavelength of the X-rays (*λ *= 1.54178 Å), *θ* is the Bragg diffraction angle, and *β* is the full width at half maximum (FWHM). The average crystallite size *d* before and after reaction calculated from the diffraction peaks was found to be approximately 8.013 and 9.158 nm, respectively. After the reaction, the average grain size of the nickel powder increased by 1.145 nm.

The diffraction peaks of nickel before and after the reaction are at 2*θ* = 44.48°, 51.78° and 76.46°, which correspond to the (111), (200) and (220) planes of the nickel, respectively. The diffraction peak 2*θ* (°) before and after the reaction did not change substantially, but the peak intensity after the reaction increased slightly, and the baseline shift of the FWHM (half full width) is small, indicating that the grain agglomeration diameter is increased.

### 3.2. Determination of the nickel powder by scanning electron microscopy

**Figure 3.**
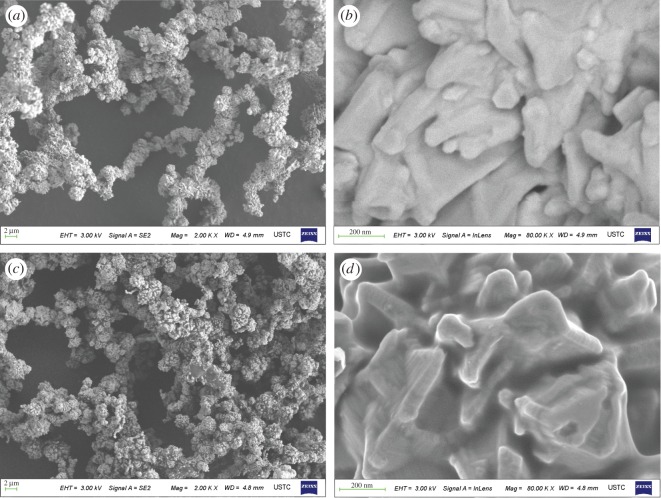
(*a–d*) SEM images of the nickel powder catalyst in the first stage before and after the reaction.

Figure 3*a*–*d* are SEM images of the nickel powder in the first stage before and after the reaction, respectively. As shown in the figure, when the nickel particles are of thickness of 2 μm before the reaction, nickel powder particles were in a linear aggregation state; compared with the spent nickel from the used granular aggregates are found to have a linear appearance. The results show that the linear morphology of the nickel powder has no change, but the agglomeration between the nickel particles is obviously enhanced, the linear nickel powder has a polycrystalline structure and nanometre thorns of single-crystal structure were found.

### 3.3. Powder X-ray diffraction determination of the NiO*_x_*–MoO*_y_*/SiO_2_ catalyst

In the microwave reactor, the crystal structure and phase analysis of the NiO*_x_*–MoO*_y_*/SiO_2_ sample in the second stage of the reactor was performed by powder XRD.

**Figure 4.**
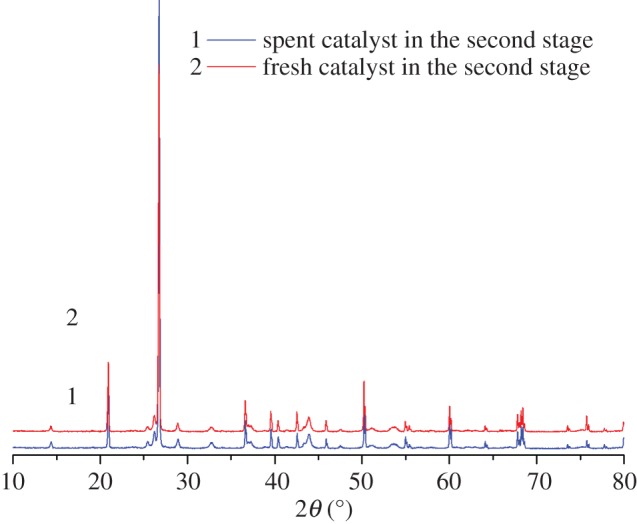
XRD patterns of the NiO*_x_*–MoO*_y_*/SiO_2_ catalysts in the second stage.

As seen from the figure, the characteristic peaks of the fresh catalyst (sample 2) at 2*θ* = 44.0° and 76.0° are attributed to the reduced Ni, and the characteristic peaks at 37.0° and 43.0° are attributed to the oxidation state NiO [22,23]. The characteristic peaks at 2*θ* = 36.6° and 25.5° belong to MoO_3_. The characteristic peaks of NiMoO_4_ in the catalyst are 2*θ* = 14.4°, 25.5°, 26.7°, 28.9°, 32.7°, 43.9° and 47.5°; among them, the characteristic peak of 26.7° is the strongest, indicating that the surface of NiMoO_4_ produces poor crystal form. The characteristic peaks appearing at 2*θ* = 25.5°, 28.9°, 32.7°, 43.9° and 47.5° are attributed to Ni–Mo/SiO_2_ [24]. Compared with the fresh catalyst, the characteristic peaks of the spent catalyst (sample 1) at 2*θ* = 76.0° are found to be attributed to the reduced Ni, and its peak intensity decreased significantly, showing that the reduced Ni metal in the NiO*_x_*–MoO*_y_*/SiO_2_ catalyst is involved in the reaction, and the active sites are partially covered. The peak intensity of the spent catalyst at 2*θ* = 26.7° is significantly higher than that of the fresh catalyst; this observation is attributed to the surface of NiMoO_4_ producing a poor crystal form, indicating that the reaction on the surface of NiMoO_4_ crystal form have an impact. By contrast, the characteristic peaks and peak intensity of NiO and MoO_3_ have almost no change, indicating that they have corresponding catalytic stability in ethylene dimerization. Powder XRD patterns of the as-prepared NiMoO_4_/SiO_2_ NPs shown in figure 4 were indexed to NiMoO_4_ according to the JCPDS database no. 33-0948.

### 3.4. Determination of the NiO*_x_*–MoO*_y_*/SiO_2_ catalyst by scanning electron microscopy

**Figure 5.**
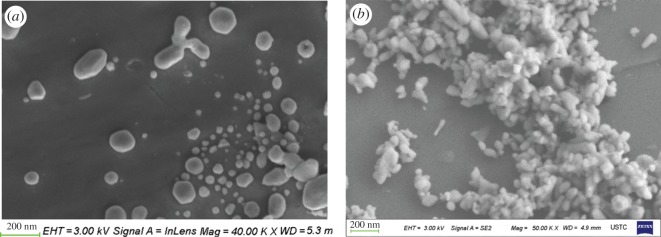
(*a,b*) SEM images of the NiO*_x_*–MoO*_y_*/SiO_2_ catalyst in the second stage.

Figure 5*a* shows the SEM images of the NiO*_x_*–MoO*_y_* /SiO_2_ catalyst in the second stage of the reactor; it can be seen from the figure that, at 200 nm, the fresh catalyst NiO*_x_*–MoO*_y_* /SiO_2_ is elliptical microspheres with macroscopic structure, and the edge is partially transparent, with a typical silica microsphere structure [25], indicating that the formation of the catalyst did not change the macroscopic structure of silicon oxide. Figure 5*b* shows an SEM image of NiO*_x_*–MoO*_y_*/SiO_2_ after the second stage of the reaction. Before the reaction, the spherical particles were less than 200 nm, the particle size of the catalyst decreased after the reaction, and the appearance showed obvious agglomeration.

### 3.5. Raman spectra

**Figure 6.**
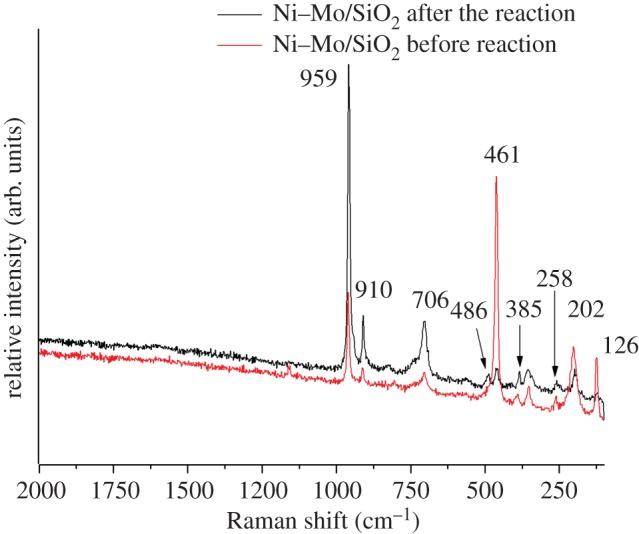
Raman spectra of the NiO*_x_*–MoO*_y_*/SiO_2_ catalyst in the second stage.

Figure 6 shows the Raman spectra of the NiO*_x_*–MoO*_y_*_(*x*=0–1,*y*=0–3)_/SiO_2_ catalyst in the second stage of the reactor. The intensive peak at 959 cm^−1^, two strong maxima at 910 and 706 cm^−1^ and a group of weak peaks at 486, 384 and 258 cm^−1^ denote the stoichiometric α-phase NiMoO_4_. The peaks at 959 and 910 cm^−1^ correspond to the symmetric and asymmetric stretching modes of the terminal Mo=O bond, and the Ni–O–Mo symmetric stretch is responsible for the peak at 706 cm^−1^. The peaks at 486 and 384 cm^−1^ have been ascribed to the bending mode of Mo–O, and the peak at 258 cm^−1^ is attributed to the deformation mode of Mo–O–Mo. This result is consistent with results reported in the literature [26,27] and is in agreement with the XRD results. After the reaction, the peak intensity of the Mo=O band at 959, 910 and 706 cm^−1^ decreased significantly, indicating that Mo=O was involved in the ethylene oligomerization reaction. A new band appeared at 461 cm^−1^, and the peak intensity is strong, which could be attributed to the reduced surface molybdenum oxide species [28]. Therefore, the Raman analysis results show that the NiO*_x_*–MoO*_y_*_(*x*=0–1,*y*=0–3)_ /SiO_2_ catalyst has the characteristic peaks of α-NiMoO_4._ After the reaction, the increase of the characteristic peak intensities of 959, 910 and 706 cm^−1^ of the α-nickel molybdate shows that the large amount of α-nickel molybdate formed leads to the inhibition of the ethylene oligomerization reaction. The intensity of the characteristic reduction peak based on 461 cm^−1^ is related to the surface reduction property of molybdenum oxide after the reaction decreased significantly. This result shows that the formation of reduced molybdenum is beneficial to the ethylene oligomerization and the formation of butene.

### 3.6. Gas reactants and liquid products

In the first stage of the reactor, under the action of microwave heating and pure nickel powder, methane dehydrogenation coupling to produce ethylene was performed. The hydrogen atmosphere inhibited the further dehydrogenation of ethylene because the reaction gas effluent did not detect acetylene. In the second stage of the reactor, under the action of microwave heating and nickel–molybdenum/SiO_2_ catalyst, the intermediate ethylene was coupled to produce butene, which contained 89.44%, and the rest are ethanol, acetic acid, butenol and butyric acid, with contents of 1.0–3.0 wt%. According to GC/EI/MS analysis of the liquid products, in addition to high levels of butene, there are trace amounts of oxygen mixed into the reaction system. Thus, butene is possibly partially oxidized to ethanol, acetic acid, butenol and butyric acid.

Table 1 shows the feed composition and the total flow rate of mixed gas. As the feed gas CH_4_/H_2_ ratio increases from 1 : 5 v/v to 1 : 4 v/v, that is, the methane flow rate does not change, the hydrogen flow rate reduced, and the methane conversion decreased slowly from 73.2 to 71.0%. When the total gas flow rate is 60 ml min^−1^, the volume ratio of methane to hydrogen is 1 : 5, the selectivity of butene is 99.0%, and the conversion rate of methane is 73.2%; this may be caused by sufficient hydrogen partial pressure, resulting in the high reaction conversion rate. However, with the hydrogen flow decreases, such as the volume ratio of methane to hydrogen reduced from 1 : 4 to 1 : 3, because of the reduced partial pressure of hydrogen, a slight decrease in the conversion of methane from 71.0 to 67.2% occurs, implying that it is necessary to maintain a certain partial pressure of hydrogen. This outcome may occur because the methane conversion reaction rate is not only related to the velocity constant, the methane partial pressure and the percentage of active sites but also to the partial pressure of the hydrogen. We believe that the amount of H_2_ also directly determines the amount of regenerated H–Ni–O–H catalyst *in situ*. The obtainable yield depends on the catalytic cycle. In fact, hydrogen is not only a reaction raw material but also an inhibitor of coke and acetylene and plays the role of carrier gas, the latter promoting the flow of reactants through the catalyst layer; as a result, the amount of hydrogen should be excessive. The results also show that, when the methane content is low and the hydrogen is in excess, the conversion of methane and the selectivity of butene depend only on the activity of nickel and NiO*_x_*–MoO*_y_*/SiO_2_ composite catalysts and are independent of the hydrogen partial pressure.

**Table 1.** Feed composition and total flow rate. Note: 700 w, 0.1–0.2Mpa, NTP, 30 min.

CH_4_ : H_2_ (v/v)	flow rate (ml min^−1^)	conversion CH_4_ (%)	selectivity C_4_H_8_ (%)
1 : 5	60	73.2	99
1 : 4	60	71.0	99
1 : 3	60	67.2	99

### 3.7. Gas chromatography–thermal conductivity detector determination of gas-phase reactants

**Figure 7.**
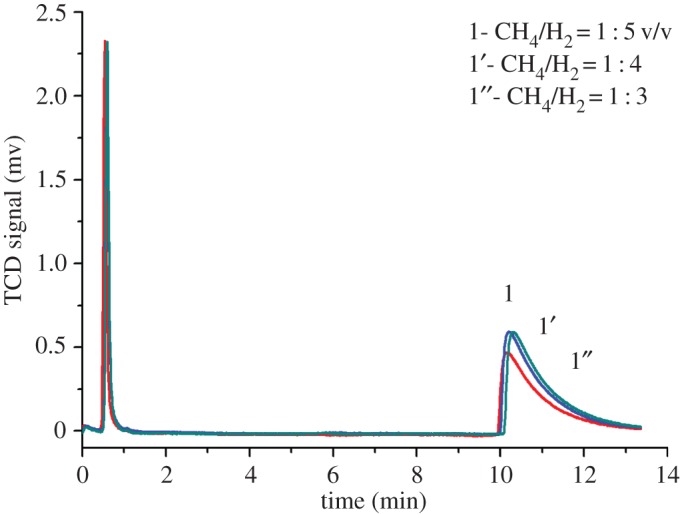
The GC-TCD analysis of the total products of the reaction gas phase. (Note: the power is 700 W; CH_4_/H_2_ = 1 : 3–5 v/v, 0.1–0.2 MPa, the conversion rate of methane: $X_{{\mathrm{CH}}_{4}}$(%) = [(moles of CH_4_ consumed)/(moles of CH_4_ introduction)] × 100; the selectivity of butene: $X_{C_{4}H_{8}}$(%) = [(moles of C_4_H_8_ produced)/(moles of CH_4_ consumed)] × 100).

Figure 7 shows the gas chromatogram of the total reaction gas product before condensation. As seen from the figure, the methane content was 26.8% at the retention time of 0.5 min, and when the retention time was 10.5 min, the butene content was 73.2%; see the GC–MS analysis. Therefore, the methane conversion was greater than 73.2%, and no further larger impurity peaks were observed from the gas-phase reaction effluent.

### 3.8. Determination of liquid products by gas chromatography/electron impact ionization/mass spectrometry

**Figure 8.**
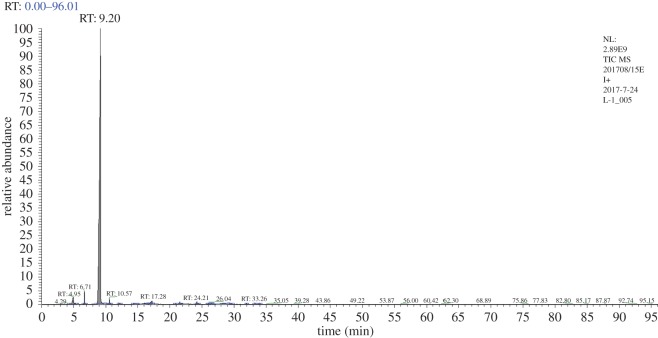
GC/MS mass spectrometry analysis of the total ion flow for liquid product.

The reaction process collects the condensed liquid; its GC/MS mass spectrum of total ion current diagram is shown in figure 8. The maximum peak time is 9.2 min, and its ion content is 89.44%; the peak time of the remaining ions was 4.95, 6.71, 10.57, 17.28 and 28.4 min, with the corresponding fragment contents of 3.02, 0.92, 0.88, 1.31 and 1.33%, respectively.

Table 2 shows the chromatographic ion outflow peak time; the content of the peak was greater than 1.0 wt% for retention times of 4.95, 9.2, 17.28 and 28.4 min, and the remaining 1.0% or less had a total of 4.90%.

**Table 2.** Chromatographic ion flow list.

apex RT	start RT	end RT	%area	%height
4.95	4.28	5.54	3.02	2.36
6.71	6.65	6.88	0.92	4.19
9.20	8.51	9.93	89.44	86.17
10.57	10.45	10.82	0.88	2.03
12.06	12.01	12.4	0.25	0.47
14.34	14.19	14.49	0.09	0.18
16.06	14.99	16.58	0.84	0.34
17.28	16.67	17.33	1.31	0.93
20.89	20.8	21.0	0.07	0.17
21.57	21.51	21.8	0.37	0.69
24.21	24.06	24.52	0.55	0.76
26.04	25.86	26.18	0.24	0.43
26.43	26.36	26.53	0.10	0.16
28.40	26.70	29.09	1.33	0.30
29.61	29.39	29.86	0.15	0.15
31.98	31.89	32.04	0.07	0.17
33.26	33.11	33.55	0.29	0.38
33.96	33.86	34.15	0.08	0.13

**Figure 9.**
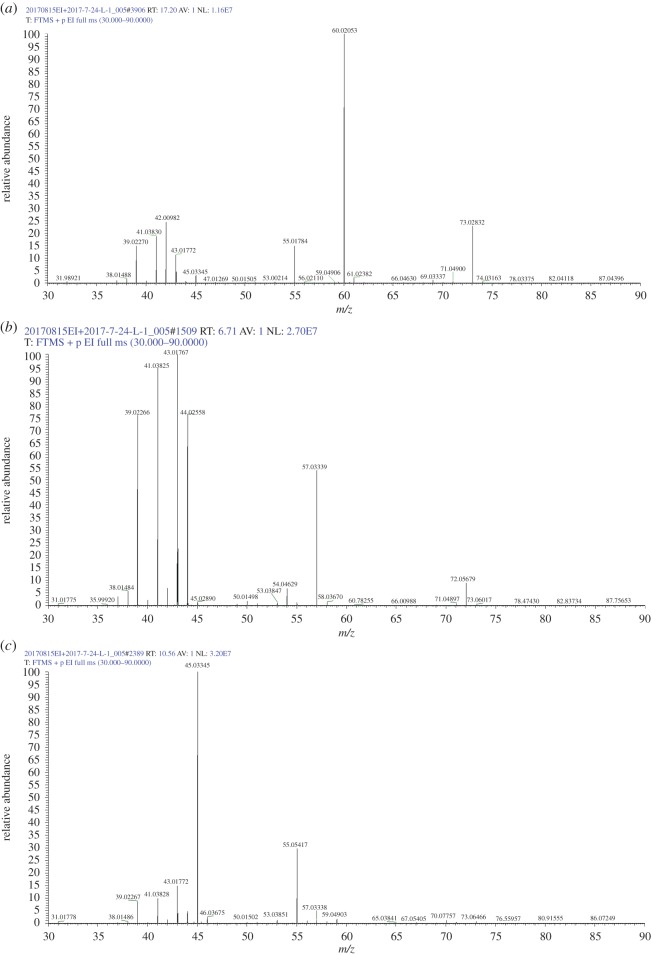

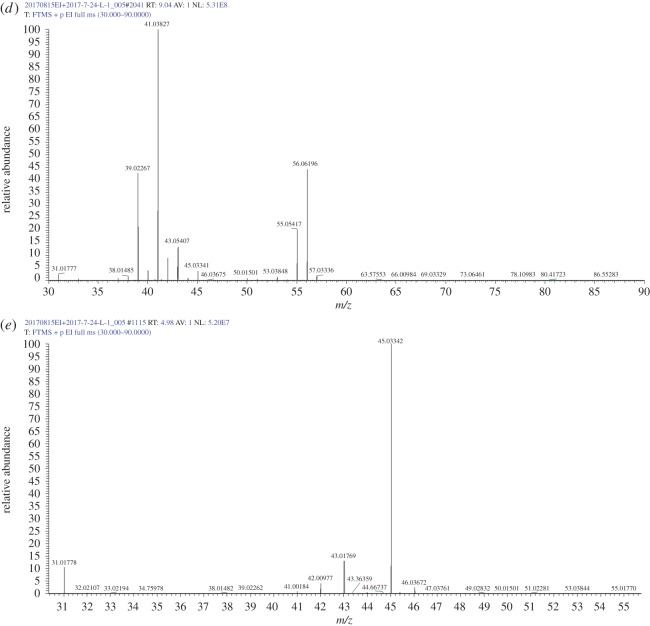
Mass–charge ratio (*m/z*) of GC/EI/MS: (*a–d*) *m/z* 30–90 and (*e*) *m/z* 30–55.

Figure 9*a* shows the mass–charge ratio; its *m/z* interval was 30–90, and the relative abundance of large ion molecules is as follows: *m/z* 55.0, *m/z* 45, *m/z* 60.02 and *m/z* 73. 03. The former is butyric acid debris, with chemical formula of acetic acid (C_2_H_4_O_2_); the latter is butyrate (removal of methyl, (CH_3_) debris C_3_H_5_O_2_. Figure 9*b* shows the *m/z* mass-to-charge ratio; the *m/z* interval is 30–90, and the relative abundance of molecular ions is as follows: *m/z* 39, *m/z* 41, *m/z* 43, *m/z* 44, *m/z* 57.03 and *m/z* 72. Figure 9*c* shows the mass-to-charge ratio, and its interval is 30–90. The relative abundances of *m/z* 53, *m/z* 41, *m/z* 43 and *m/z* 45 correspond to ethanol ion fragments, and those of *m/z* 53.03, *m/z* 55.05 and *m/z* 57.03 correspond to the butene debris of ${\text{C}}_{4}{\text{H}}_{7}^{+}$ and ${\text{C}}_{4}{\text{H}}_{5}^{+}$ and C_3_H_5_O. The *m/z* 57.06 corresponds to the butene isotopes C_4_H_9_^+^. Figure 9*d* shows the mass-to-charge ratio, and its *m/z* interval is 30–90. The relative abundance of molecular ions are *m/z* 56.06, *m/z* 55.05, *m/z* 45, *m/z* 43, *m/z* 41 and *m/z* 39, where *m/z* 56.06 corresponds to butene, *m/z* 55.05 corresponds to butene debris, *m/z* 45 corresponds to ethanol debris and *m/z* 41 corresponds to propylene debris. Figure 9*e* shows the mass-to-charge ratio, and its *m/z* interval is 30–55; the relative abundance of molecular ion of *m/z* 43.02 and *m/z* 45.03 correspond to ethanol fragments.

### 3.9. Methane non-oxidative dehydrogenation coupling into butene mechanism

**Scheme 1.**
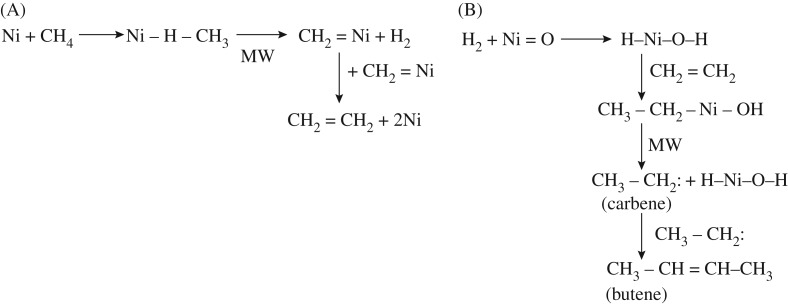
(A,B) Shows the catalytic dehydrogenation of methane to prepare butene by oligomerization of ethylene. MW, microwave.

**Scheme 2.**
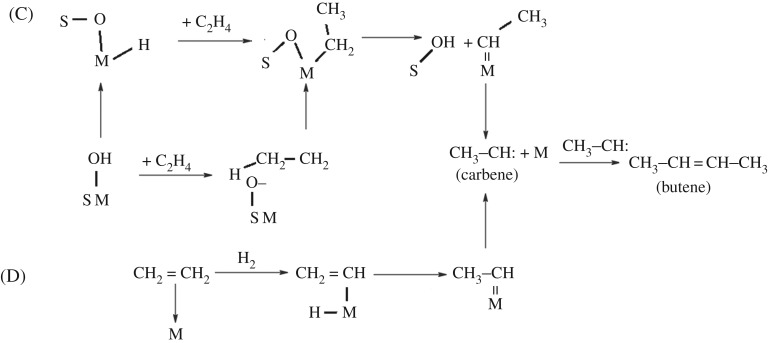
(C,D) Shows the possible routes of the ethylene oligomerization to produce butene. M represents the catalytic active site of Ni or Mo in NiMoO_4_ salt and S represents the silica support.

**Scheme 3.**
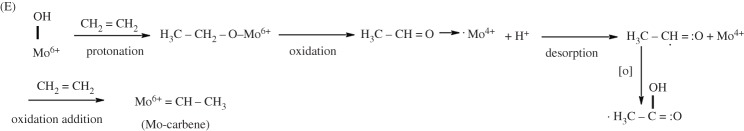
(E) Shows the proposed route for the formation of the small molecules containing oxygen starting from a Mo(VI) site upon interaction with two ethylene molecules.

The non-oxidative dehydrogenation of methane to produce butene is extremely complex. The mechanism involves the nickel powder in the first stage of the reaction and the NiO*_x_*–MoO*_y_*/SiO_2_ composite in the second stage of the reaction. Under the combined action of nickel metal and NiO*_x_*–MoO*_y_*/SiO_2_ catalyst and microwave heating, unlike conventional microwave plasma methane conversion [29–33], the possible mechanism of methane non-oxidative dehydrogenation oligomerization in this reaction is shown in schemes 1–3.

#### 3.9.1. Path (A)

In the first stage of the reactor, the nickel powder absorbs methane by polarization of the C–H bond. Under the microwave action, methane is transformed into carbene (H_2_C:) and then ethylene (CH_2_=CH_2_). The likely reactions are absorption of methane by Ni metal, according to equation (3.2):

3.2 $$\text{Ni}+\text{C}{\text{H}}_{4}\to \text{Ni}-\text{H}-\text{C}{\text{H}}_{3}(\text{C}-\text{H bond polarization}).$$

At this point, the carbon–hydrogen bond is polarized, and under the action of microwave, the linkage breaks and the dissociation of hydrogen molecules occur. See reaction formula (3.3):

3.3 $$\text{Ni}-\text{H}-\text{C}{\text{H}}_{3}+\text{microwave}\to \text{C}{\text{H}}_{2}=\text{Ni}+{\text{H}}_{2}.$$

According to formula (3.4), the formation of CH_2_=CH_2_ occurred:

3.4 $$2\text{C}{\text{H}}_{2}=\text{Ni}\to \text{C}{\text{H}}_{2}=\text{C}{\text{H}}_{2}+2\text{Ni}.$$

#### 3.9.2. Path (B)

In the second stage of the reactor, in the presence of H_2_, nickel oxide reacts with hydrogen according to reaction formula (3.5):

3.5 $${\text{H}}_{2}+\text{Ni}=\text{O}\to \text{H}-\text{Ni}-\text{O}-\text{H}.$$

Next, ethylene and H–Ni–O–H undergo an endothermic reaction according to reaction formula (3.6):

3.6 $$\text{C}{\text{H}}_{2}=\text{C}{\text{H}}_{2}+\text{H}-\text{Ni}-\text{O}-\text{H}\to \text{C}{\text{H}}_{3}-\text{C}{\text{H}}_{2}-\text{Ni}-\text{OH}.$$

Under the action of microwave, the carbene was obtained, see formula (3.7):

3.7 $$\text{C}{\text{H}}_{3}-\text{C}{\text{H}}_{2}-\text{NiOH}+\text{MW}\to \text{C}{\text{H}}_{3}-\text{CH}:(\text{carbine})+\text{H}-\text{Ni}-\text{O}-\text{H}.$$

The association of equations (3.6) and (3.7) form a catalytic cycle.

Next, coupling of two molecules of carbene result in a molecule of butane; see formula (3.8):

3.8 $$2\text{C}{\text{H}}_{3}-\text{CH}:(\text{carbene})\to \text{C}{\text{H}}_{3}-\text{CH}=\text{CH}-\text{C}{\text{H}}_{3}.$$

In the second stage, the NiMoO_4_/SiO_2_ catalyst contains NiO and MoO_3_. Under the action of hydrogen, H–Ni–OH is formed (3.5). Meeting the ethylene outflow from the first stage of the tube, the catalyst forms an intermediate CH_3_–CH_2_–Ni–OH (3.6). Under microwave action, the catalyst can either take off a hydrogen from ethyl group and form a methyl-carbene (CH_3_CH_2_–Ni–OH + MW → H–Ni–OH +CH_3_**–**CH:) (3.7) and then butene, see formula (3.8), which is a catalytic reaction.

#### 3.9.3. Path (C)

In the second stage, one of the possible pathways for the formation of butene from ethylene is (C) [34–36]. From nickel molybdate and carrier silica, with a hydroxyl group of nickel or molybdenum, the hydrogen on the hydroxyl group is rearranged within the molecule on the metal M, as shown in reaction formula (3.9), the following M represents nickel or molybdenum derived from nickel molybdate, and S represents a silica support:

3.9 HO−S−M→S−O−M−H.

The hydrogenated metal (S–O–M–H) undergoes a protonation reaction with ethylene to form a complex (CH_3_–CH_2_–M–O–S); see (3.10):

3.10 $$\text{S}-\text{O}-\text{M}-\text{H}+{\text{C}}_{2}{\text{H}}_{4}\to \text{C}{\text{H}}_{3}-\text{C}{\text{H}}_{2}-\text{M}-\text{O}-\text{S}.$$

The hydroxyl compound (HO–S–M) can also react directly with ethylene to form a carbon positive ion $\left({\text{H}}_{3}\text{C}-{\text{CH}}_{2}^{+}\right)$ and an oxygen anion (M–S–O^−^); see formula (3.11):

3.11 $$\text{HO}-\text{S}-\text{M}+{\text{C}}_{2}{\text{H}}_{4}\to [\text{C}{\text{H}}_{3}-\text{C}{{\text{H}}_{2}}^{+}][{\;}^{-}\text{O}-\text{S}-\text{M}],$$

carbon positive ion $\left({\text{H}}_{3}\text{C}-{\text{CH}}_{2}^{+}\right)$ and oxygen anion (M–S–O^−^) pairs forming an intra-molecular rearrangement, and then the complex (S–O–M–CH_2_–CH_3_) was obtained; see formula (3.12):

3.12 $$[\text{C}{\text{H}}_{3}-{\text{CH}}_{2}^{+}][{\;}^{-}\text{O}-\text{S}-\text{M}]\to \text{S}-\text{O}-\text{M}-\text{C}{\text{H}}_{2}-\text{C}{\text{H}}_{3}.$$

When the complex (S–O–M–CH_2_–CH_3_) is subjected to microwave and heating, it will undergo a dissociation reaction to produce hydroxyl silicon (S–OH) and metal carbene (M = CH–CH_3_); see formula (3.13):

3.13 $$\text{S}-\text{O}-\text{M}-\text{C}{\text{H}}_{2}-\text{C}{\text{H}}_{3}\to \text{S}-\text{OH}+\text{M}=\text{CH}-\text{C}{\text{H}}_{3}.$$

When the metal carbene (M=CH–CH_3_) is subjected to microwave and heat, it decomposes into metal M and carbine; see formula (3.14):

3.14 $$\text{M}=\text{CH}-\text{C}{\text{H}}_{3}\to \text{C}{\text{H}}_{3}-\text{CH}:+\text{M}.$$

According to formula (3.15), two molecules carbene polymerization reaction will produce butene:

3.15 $$\text{C}{\text{H}}_{3}-\text{CH}:+\text{C}{\text{H}}_{3}-\text{CH}:\to \text{C}{\text{H}}_{3}-\text{CH}=\text{CH}-\text{C}{\text{H}}_{3}.$$

#### 3.9.4. Path (D)

The possible path for the formation of butene from ethylene is (D) [34,37], and nickel or molybdenum from the nickel molybdate salt can also be combined with ethylene to form a complex [CH_2_=CH][M–H]; see formula (3.16):

3.16 $$[\text{C}{\text{H}}_{2}=\text{C}{\text{H}}_{2}][\text{M}]\to [\text{ C}{\text{H}}_{2}=\text{CH}][\text{M}-\text{H}].$$

The complex [CH_2_=CH][M–H] can undergo an intra-molecular hydrogen rearrangement to produce a more stable complex (CH_3_–CH=M); see formula (3.17):

3.17 $$[\text{C}{\text{H}}_{2}=\text{CH}][\text{M}-\text{H}]\to \text{C}{\text{H}}_{3}-\text{CH}=\text{M}.$$

When the complex (CH_3_–CH=M) is decomposed, a molecule carbene and M are generated, as shown in formula (3.18):

3.18 $$\text{C}{\text{H}}_{3}-\text{CH}=\text{M}\to \text{C}{\text{H}}_{3}-\text{CH}:(\text{carbene})+\text{M}.$$

When the two molecules carbene coupling, one molecule butene is obtained; see formula (3.19):

3.19 $$\text{C}{\text{H}}_{3}-\text{CH}:+\text{C}{\text{H}}_{3}-\text{CH}:\to \text{C}{\text{H}}_{3}-\text{CH}=\text{CH}-\text{C}{\text{H}}_{3}(\text{butene}).$$

#### 3.9.5. Path (E)

(E) is one possible path for the formation of butene from ethylene [38]. From nickel molybdate, the hexavalent molybdenum with hydroxyl groups can also protonize ethylene, generating the complex (H_3_C–CH_2_–O– Mo^6+^); see formula (3.20):

3.20 $$\text{HO}-\text{M}{\text{o}}^{6+}+\text{C}{\text{H}}_{2}=\text{C}{\text{H}}_{2}\to {\text{H}}_{3}\text{C}-\text{C}{\text{H}}_{2}-\text{O}-\text{M}{\text{o}}^{6}.$$

According to formula (3.21), the complex (H_3_C–CH_2_–O– Mo^6+^) is subjected to trace oxygen to remove a proton, forming the complex (H_3_C–CH=O– Mo^4+^), where the complex is oxidized, and the hexavalent molybdenum is reduced:

3.21 $${\text{H}}_{3}\text{C}-\text{C}{\text{H}}_{2}-\text{O}-\text{M}{\text{o}}^{6+}+\text{oxidation}\to {\text{H}}_{3}\text{C}-\text{CH}=\text{O}-\text{M}{\text{o}}^{4+}+\text{H}.$$

The complex (H_3_C–CH=O–Mo^4+^) is unstable, easy to dissociate, and generates (H_3_C–CH=O) and Mo^4+^ metal ions; see formula (3.22):

3.22 $${\text{H}}_{3}\text{C}-\text{CH}=\text{O}-\text{M}{\text{o}}^{4+}\to {\text{H}}_{3}\text{C}-\text{CH}=\text{O}+\text{M}{\text{o}}^{4+}.$$

According to formula (3.23), acetaldehyde is extremely unstable and easily produces acetic acid:

3.23 $${\text{H}}_{3}\text{C}-\text{CH}=\text{O}+[\text{O}]\to {\text{H}}_{3}\text{C}-\text{COOH}.$$

While tetravalent molybdenum and ethylene continue to oxidize and react, Mo–carbene (Mo^6+^=CH–CH_3_) will be obtained:

3.24 $$\text{M}{\text{o}}^{4+}+\text{C}{\text{H}}_{2}=\text{C}{\text{H}}_{2}\to \text{M}{\text{o}}^{6+}=\text{CH}-\text{C}{\text{H}}_{3}(\text{Mo}-\text{carbene}).$$

Mo–carbene easily dissociates into carbene and molybdenum:

3.25 $$\text{M}{\text{o}}^{6+}=\text{CH}-\text{C}{\text{H}}_{3}(\text{Mo}-\text{carbene})\to \text{M}{\text{o}}^{6+}+\text{C}{\text{H}}_{3}-\text{CH}:\;(\text{carbene}).$$

Mo–carbene can also give its OH by forming ethanol (CH_3_CH_2_–Ni–OH + MW → CH_3_CH_2_OH + Ni) in the second stage of the reactor. Ethanol can also generate all the oxygenated impurities observed in the condensed liquid, for example C_2_H_4_O_2_ and C_4_H_8_O.

## 4. Conclusion

Under the condition of 0.1–0.2 MPa, methane was directly converted into butene liquid fuel with high selectivity through a microwave-induced catalytic reaction. The results show that the use of nickel powder and NiO*_x_*–MoO*_y_*/SiO_2_ in the two-stage fixed-bed reactor under microwave heating. Raman analysis showed that there were α-NiMoO_4_ characteristic peaks in the catalyst NiO*_x_*–MoO*_y_*/SiO_2_, and nickel molybdate will inhibit the polymerization of ethylene to produce butene. The excess hydrogen in the methane–hydrogen mixture can delay carbon deposition. The methane conversion was 73.2%, and the methane selectivity to butene was 99.0%. The results of GC/EI/MS ion chromatographic analysis showed that the liquid fuel produced by methane dehydrogenation contained 89.44% butene, and the rest was ethanol, acetic acid, butenol and butyric acid of contents of 1.0–3.0 wt%.

## Data accessibility

This article has no additional data.
